# Quality of life and patient‐reported toxicities in patients with advanced Merkel cell carcinoma treated with combined nivolumab and ipilimumab with or without stereotactic body radiation therapy

**DOI:** 10.1002/cam4.7464

**Published:** 2024-07-17

**Authors:** Aasha I. Hoogland, Andrew S. Brohl, Brent J. Small, Lauren Michael, Evan Wuthrick, Zeynep Eroglu, Dukagjin Blakaj, Claire Verschraegen, Nikhil I. Khushalani, Heather S. L. Jim, Sungjune Kim

**Affiliations:** ^1^ Department of Health Outcomes and Behavior Moffitt Cancer Center Tampa Florida USA; ^2^ Department of Cutaneous Oncology Moffitt Cancer Center Tampa Florida USA; ^3^ School of Aging Studies University of South Florida Tampa Florida USA; ^4^ Department of Radiation Oncology Moffitt Cancer Center Tampa Florida USA; ^5^ Department of Radiation Oncology Ohio State University Columbus Ohio USA; ^6^ Department of Cutaneous Oncology Ohio State University Columbus Ohio USA; ^7^ Department of Radiation Oncology Mayo Clinic Jacksonville Florida USA

**Keywords:** immune checkpoint inhibitor therapy, Merkel cell carcinoma, patient‐reported outcomes, quality of life

## Abstract

**Background:**

Merkel cell carcinoma is a rare skin cancer associated with poor survival. Based on a previous Phase II trial of adults with advanced Merkel cell carcinoma by Kim and colleagues (2022), there is now a strong rationale for combination therapy (i.e., nivolumab and ipilimumab) to become a treatment option for patients with advanced Merkel cell carcinoma. The goal of this paper was to report on the secondary outcome of quality of life (QOL) among patients on this trial.

**Methods:**

Patients receiving combined nivolumab and ipilimumab, with or without stereotactic body radiation therapy (SBRT), completed the European Organisation for Research and Treatment of Cancer (EORTC) QLQ‐C30 prior to starting treatment and every 2 weeks thereafter. Changes in QOL during treatment and post‐treatment were evaluated using piecewise random‐effects mixed models. Exploratory analyses compared changes in QOL between study arms. The original trial was registered with ClinicalTrials.gov (NCT03071406).

**Results:**

Study participants (*n* = 50) reported no changes in overall QOL (*p*s > 0.05), but emotional functioning improved during treatment (*p* = 0.01). Cognitive and social functioning worsened post‐treatment (*p*s < 0.01). In general, patients treated with combination therapy only (*n* = 25) reported no change in QOL over time, whereas patients also treated with SBRT (*n* = 25) consistently demonstrated worsening QOL post‐treatment.

**Conclusion:**

QOL is generally preserved in patients treated with combination therapy, but the addition of SBRT may worsen QOL. Combined with clinical efficacy data published previously, results support the use of combination therapy with nivolumab and ipilimumab as a treatment option for patients with advanced Merkel cell carcinoma.

## 1 INTRODUCTION

Merkel cell carcinoma is a rare, deadly skin cancer associated with poor survival rates. Current standard of care for patients diagnosed with Merkel cell carcinoma includes single‐agent monotherapies, including immune checkpoint inhibitors such as the programmed death protein 1 (PD‐1) monoclonal antibody pembrolizumab and the programmed death ligand 1 (PD‐L1) monoclonal antibody avelumab. Patients treated with pembrolizumab or avelumab demonstrate objective response rates ranging from 33% to 58%.^1^, ^2^, ^3^, ^4^, ^5^ However, many patients remain resistant to these monotherapies. Other safe and efficacious treatment options are needed.

A recent Phase II trial from Kim and colleagues^6^ suggests promising efficacy of combined nivolumab and ipilimumab particularly among a subgroup of patients who had not been previously treated with immune checkpoint inhibitors (ICIs). In this trial, patients were randomized to receive combination therapy with nivolumab and ipilimumab only, or combination therapy with nivolumab and ipilimumab with the addition of stereotactic body radiation therapy (SBRT). There was no statistically significant difference in objective response rates between treatment groups; 72% patients treated with combined nivolumab and ipilimumab demonstrated an objective response to treatment, compared to 52% of patients who were also treated with SBRT. Stratified subgroup analyses revealed that 100% of evaluable patients who were ICI‐naïve (22 out of 50 patients) demonstrated an objective response to treatment, with 41% demonstrating a complete response. In contrast, 31% of patients who previously received pembrolizumab or avelumab had an objective response to treatment, with only 15% having a complete response. Nearly all (90%) of the 50 patients recruited to the study demonstrated clinician‐rated treatment‐related adverse events. Consistent with FDA guidelines,^7^ we report on patient‐reported quality of life (QOL) in the patients who participated in the Kim et al.^6^ trial.

Among patients diagnosed with Merkel cell carcinoma, studies examining patient‐reported QOL have generally focused on patients treated with single‐agent monotherapies. Patients treated with single‐agent nivolumab reported generally stable QOL during treatment, with marginal improvements in emotional well‐being.^8^ Patients diagnosed with cutaneous squamous cell carcinoma, a cutaneous malignancy less aggressive than Merkel cell carcinoma, and treated with single‐agent pembrolizumab, also reported stable QOL during treatment.^9^ Among patients treated with single‐agent avelumab, chemorefractory patients who responded to treatment reported no change in QOL, whereas patients with disease progression reported worsening QOL during treatment (*p* = 0.02).^10^ Treatment‐naïve patients treated with single‐agent avelumab reported no change in overall QOL, but significant improvements in emotional well‐being (*p* < 0.001).^11^ In addition, treatment‐naïve patients demonstrating a disease response to avelumab reported QOL scores that, on average, were more than 4 points better (i.e., the minimally important difference on the total FACT‐G)^12^ than patients whose disease progressed. These findings suggest that QOL may remain stable or improve over time in patients treated with single‐agent monotherapies, but may decline among patients who do not respond to treatment. Furthermore, studies examining PROs in patients treated with combination ICIs suggest that patients report stable QOL after treatment initiation, similar to or better than patients treated with single‐agent immunotherapies.^13^, ^14^, ^15^ However, QOL in patients diagnosed with Merkel cell carcinoma who are treated with combination ICIs is largely unknown.

This paper reports on QOL outcomes among advanced Merkel cell carcinoma patients who participated in the Phase II trial of combination nivolumab and ipilimumab with or without SBRT.^6^ In addition to the per‐protocol analyses evaluating change in QOL across all study participants, we conducted post hoc analyses to examine differences in patient‐reported QOL between the two treatment arms. Because changes in QOL can result from complex and multifactorial causes, this paper is intended to be descriptive rather than to ascribe changes in QOL to particular disease or treatment factors.

## 2 MATERIALS AND METHODS

### 2.1 Participants and procedure

Full inclusion and exclusion criteria have been published previously.^6^ Briefly, patients were eligible for participation if they were at least 18 years of age; diagnosed with unresectable, recurrent, or stage IV Merkel cell carcinoma; scheduled to receive treatment at either Moffitt Cancer Center (Florida, USA) or Ohio State University James Cancer Hospital and Solove Research Institute (Ohio, USA); determined to have an Eastern Cooperative Oncology Group (ECOG) performance status of 0 or 1; and diagnosed with at least two histologically proven measurable tumor lesions. The protocol was approved by the Advarra Institutional Review Board prior to study commencement. Participants provided written informed consent and completed questionnaires approximately every 2 weeks until disease progression, unacceptable toxicity, or withdrawal of consent. There were two planned follow‐up assessments at 30 days and 114 days after treatment completion. Information on randomization, masking, and study procedures have been published previously.^6^ This study was also registered with ClinicalTrials.gov (NCT03071406).

### 2.2 Measures

Demographic and clinical information were abstracted from participants' medical charts. Variables included age, sex, race, ECOG performance status (0 or 1), disease stage (IIIB or IV), primary tumor site, metastatic stage, number of distant metastatic sites, lactate dehydrogenase concentration (normal or elevated), prior receipt of ICI (yes/no), and prior treatment with chemotherapy (yes/no). The 30‐item European Organisation for Research and Treatment of Cancer (EORTC) QLQ‐C30^16^ was used to assess QOL. Participants completed 28 questions using a 4‐point Likert scale (1 = not at all, 4 = very much) and 2 questions on overall health and quality of life using a 7‐point Likert scale (1 = very poor, 7 = excellent). The EORTC QLQ‐C30 yields a global health status score (e.g., overall QOL) and five QOL subscales (i.e., physical, role, emotional, cognitive, and social).^17^ Select single‐item symptoms (i.e., fatigue, nausea, vomiting, dyspnea, diarrhea) were compared to previously reported clinician‐rated toxicities.^6^ Higher scores on the quality of life items and subscales indicated better QOL. Higher scores on the symptom items indicated greater symptomatology. A change of 10 points^9^, ^18^ or a group difference of 10 points^19^ on the EORTC QOL is considered clinically meaningful. Given small subgroup sizes, post hoc analyses of QOL by previous receipt of ICIs were run on overall QOL only.

### 2.3 Data analyses

Participants were included in analyses if they received at least one dose of nivolumab with ipilimumab and completed at least one questionnaire. Because of the variability in time spent in active treatment, patient‐reported outcome data collected during treatment were truncated at the third quartile, or 37 weeks (i.e., 75% of participants completed assessments through 37 weeks of active treatment, thus on‐treatment observations provided after 37 weeks were removed from analyses). Sociodemographic and clinical characteristics were described previously using frequencies and percentages.^6^ Because patient‐reported data were collected during and after treatment, piecewise random‐effects mixed models were selected, using all available data at each timepoint, with two distinct timeframes (i.e., during treatment and post‐treatment) to examine change in QOL separately in these time frames in all randomized patients. Estimated means and 95% confidence intervals were used to describe QOL in all randomized patients at four timepoints: baseline (pre‐treatment), 12 weeks after starting treatment, 30 days after completing treatment, and 114 days after completing treatment. The 12‐week timepoint was selected because of its clinical relevance (i.e., this is approximately when the first efficacy assessment was completed).

Piecewise mixed models were used to conduct exploratory analyses of group differences (i.e., combined nivolumab and ipilimumab only vs. nivolumab and ipilimumab with SBRT) in change in QOL during treatment and post‐treatment. Between‐subjects comparisons of least squares means from the piecewise mixed models were used to evaluate cross‐sectional differences in QOL by treatment arm. Similarly, piecewise mixed models and between‐subjects comparisons of least squares means were used to evaluate differences in overall QOL by prior ICI. Patient‐reported symptoms during treatment were described using frequencies and percentages and compared descriptively to previously reported^6^ physician‐rated toxicity grades per the Common Terminology Criteria for Adverse Events.^20^ For these comparisons, the greatest self‐reported severity of each symptom was compared to the highest grade adverse event reported by physicians. All statistical analyses were performed using SAS 9.4 (Cary, NC).

## 3 RESULTS

Participant characteristics (*n* = 50) have been described in detail previously.^6^ Briefly, participants were 73 years of age on average, White (100%), and most were male (78%). About half of patients had an ECOG performance status of 0 (46%), and a similar percentage had been treated previously with immune checkpoint inhibitor therapy (52%). As described in the main clinical outcomes paper, there were no significant differences in sociodemographic or clinical characteristics between the two treatment arms.^6^ At baseline, 46 (92%) patients provided data, and by the 114 day follow‐up, 19 (38%) patients provided data.

### 3.1 Quality of life

Estimated means and 95% confidence intervals for each QOL domain are presented in Table 1. Raw means and standard deviations for overall QOL, along with the number of participants providing data at each timepoint, are presented in Table 2. Parameter estimates from the piecewise random‐effects mixed models examining changes in QOL in all randomized participants together are presented in Table S1. Results indicated that emotional functioning improved during treatment (*p* = 0.01), and both cognitive functioning and social functioning worsened post‐treatment (*p*s < 0.01). By the 114‐day follow‐up, there was a clinically meaningful decline in estimated social functioning relative to baseline (i.e., −13.64 points). There were no other significant changes in QOL during treatment or post‐treatment (*p*s > 0.05).

**TABLE 1:** Estimated means and 95% confidence limits for quality of life in all randomized participants over time (*n* = 50).

	Overall quality of life	Physical functioning	Role functioning	Emotional functioning	Cognitive functioning	Social functioning
Baseline	65.66 (59.33, 71.99)	76.32 (70.44, 82.21)	72.09 (63.79, 80.38)	77.79 (72.48, 83.10)	83.54 (78.72, 88.35)	76.61 (69.60, 83.61)
12 weeks	65.44 (59.44, 71.44)	75.82 (70.12, 81.51)	71.40 (63.47, 79.33)	80.40 (75.42, 85.38)	84.64 (80.20, 89.08)	76.63 (70.07, 83.19)
30 day follow‐up	63.96 (57.72, 70.19)	74.94 (69.10, 80.77)	70.45 (62.25, 78.64)	78.20 (72.99, 83.42)	81.09 (76.38, 85.80)	73.02 (66.13, 79.90)
114 day follow‐up	59.19 (50.43, 67.95)	71.04 (63.54, 78.53)	65.83 (54.69, 76.98)	79.44 (71.80, 87.08)	74.27 (66.98, 81.55)	62.97 (52.87, 73.07)

*Note*: Data were not collected at 12 weeks exactly, but efficacy assessments are often conducted at 12 weeks, and thus, 12 weeks after treatment initiation is a clinically meaningful timepoint.

**TABLE 2:** Raw means and standard deviations for overall QOL by timepoint (*n* = 50).

Timepoint	*N*	Mean	SD
Baseline	46	64.67	27.11
49 days after treatment initiation	40	69.85	22.45
91 days after treatment initiation	34	71.81	21.22
133 days after treatment initiation	26	71.15	24.06
175 days after treatment initiation	19	72.81	22.37
217 days after treatment initiation	15	74.44	18.49
259 days after treatment initiation	14	73.81	17.86
30 day follow‐up	15	64.44	20.77
114 day follow‐up	19	67.47	25.74

**FIGURE 1:**
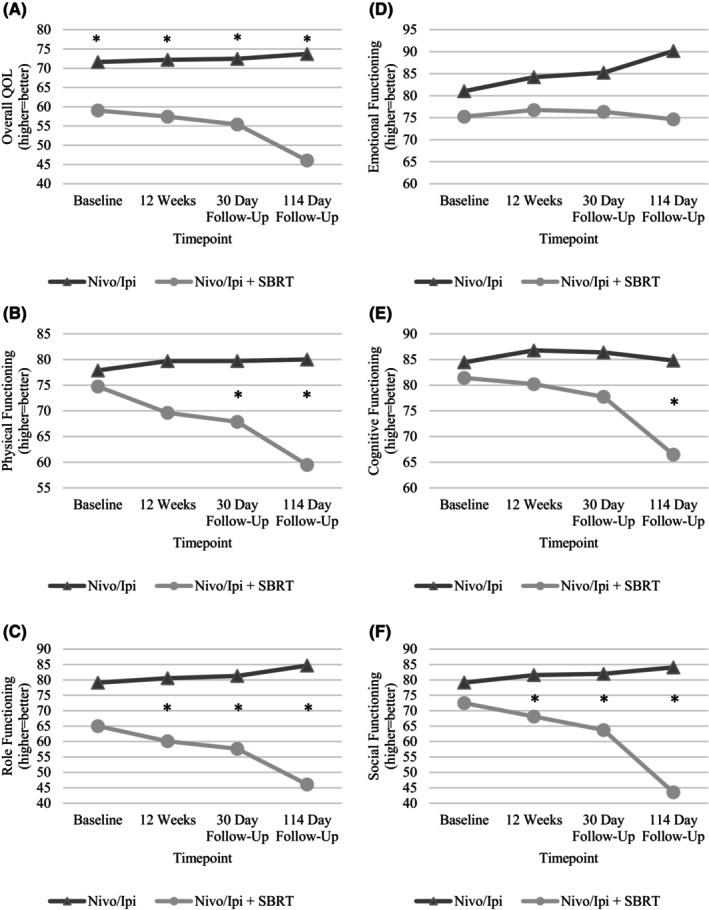
Least squares means of quality of life scores by treatment arm for: (A) overall quality of life, (B) physical functioning, (C) role functioning, (D) emotional functioning, (E) cognitive functioning, and (F) social functioning. Asterisks denote statistically significant differences by treatment arm (*p*s < 0.05).

Parameter estimates from the exploratory piecewise random‐effects mixed models examining differences in changes in QOL by treatment arm are presented in Table S2. Least squares means of QOL by treatment arm are displayed in Figure 1. There were no statistically significant differences in changes in overall QOL by treatment arm or by prior receipt of ICI (*p*s > 0.05). However, there were significant time by group interactions such that during active treatment, physical functioning (*p* < 0.001), role functioning (*p* = 0.03), and social functioning (*p* = 0.02) worsened for patients also treated with SBRT, but not patients treated with combination therapy only. Least squares means comparisons indicated significant cross‐sectional differences by treatment arm for role functioning and social functioning at 12 weeks after treatment initiation (*p*s < 0.05). Similarly, post‐treatment declines in physical functioning (*p* = 0.04), role functioning (*p* = 0.03), and social functioning (*p* < 0.001) were reported by patients also treated with SBRT, but not patients treated with combination therapy only. Least squares means comparisons indicated significant cross‐sectional differences by treatment arm for physical functioning, role functioning, and social functioning at both 30 days and 114 days after treatment completion (*p*s < 0.05). Furthermore, there were clinically relevant cross‐sectional differences by prior receipt of ICI such that patients previously treated with an ICI reported lower overall QOL (a difference of at least 10 points) than patients who were ICI‐naïve at 12 weeks and both 30 days and 114 days after treatment completion. This group difference was statistically significant at the two follow‐up timepoints (*p*s < 0.05). Patient‐reported symptom severity was consistently greater than physician‐rated symptom severity (see Table 3), for both patients treated with combination therapy only (see Table S3) and patients also treated with SBRT (see Table S4).

**TABLE 3:** Patient‐rated and clinician‐rated symptom severity (*n* = 50).

	None		A little		Quite a bit		Very much	
	Patients	Clinicians	Patients	Clinicians	Patients	Clinicians	Patients	Clinicians
Fatigue	3 (6)	18 (36)	20 (40)	31 (62)	17 (34)	1 (2)	10 (20)	0
Nausea	29 (58)	34 (68)	15 (30)	16 (32)	5 (10)	0	1 (2)	0
Vomiting	40 (80)	43 (86)	10 (20)	7 (14)	0	0	0	0
Dyspnea	22 (44)	41 (82)	16 (32)	9 (18)	9 (18)	0	3 (6)	0
Diarrhea	25 (50)	28 (56)	15 (30)	20 (40)	8 (16)	2 (4)	2 (4)	0

*Note*: “None” corresponds to “not at all” on the patient‐reported EORTC‐QLQ‐C30 and no reported adverse event on the clinician‐rated CTCAE. “A little” on the EORTC‐QLQ‐C30 corresponds to grade 1 or 2 on the CTCAE. “Quite a bit” on the EORTC‐QLQ‐C30 corresponds to grade 3 on the CTCAE. “Very much” on the EORTC‐QLQ‐C30 corresponds to grade 4 on the CTCAE.

## 4 DISCUSSION

To our knowledge, this study is the first to examine QOL in patients treated with combined nivolumab and ipilimumab for advanced Merkel cell carcinoma. Results indicated that patients treated with combined nivolumab and ipilimumab reported improved emotional functioning while on treatment, but worsened cognitive and social functioning post‐treatment. However, analyses by treatment arm indicated that patients also treated with SBRT reported worsening QOL during active treatment and post‐treatment relative to patients treated with combination therapy only.

The role of combination nivolumab plus ipilimumab in routine clinical practice for advanced Merkel cell carcinoma remains controversial as randomized trials comparing combination therapy to single agent have not been performed. From an efficacy standpoint, the Kim et al.^6^ study on which this analysis is based suggested a clinically meaningful improvement in response rate (100%, *n* = 22) over historical expectations of single‐agent anti‐PD(L)1. In contrast, in unpublished work, Bhatia et al.^21^ reported a more modest response rate of 63.6%, similar or slightly higher than historical anti‐PD(L)1 expectations in this population, using the same nivolumab plus ipilimumab combination in 33 treatment‐naïve MCC patients. Particularly given the unproven and unquantified efficacy benefit over single‐agent activity, toxicity and QOL considerations must factor into this decision. As this is the first report to examine QOL in MCC patients treated with combination nivolumab plus ipilimumab, it represents an important datapoint for clinical decision making. We observed that overall QOL does not significantly change in the overall study population receiving combination nivolumab plus ipilimumab, but that there are improvements in emotional functioning and declines in social and cognitive functioning. Furthermore, changes in overall QOL were unrelated to prior receipt of an ICI, but there were clinically relevant differences at 12 weeks and both post‐treatment timepoints such that QOL was lower in patients previously treated with an ICI as compared to patients who were ICI‐naïve.

Our results indicate that adding SBRT to combined nivolumab and ipilimumab for patients receiving treatment for Merkel cell carcinoma confers no improvement in QOL over and above treatment with combined nivolumab and ipilimumab only. In contrast, patients who were also treated with SBRT often reported worsened QOL (and worse overall QOL prior to treatment). Furthermore, while patient‐reported symptom severity was more severe than clinician‐rated symptom severity for patients in both treatment arms, fatigue in particular rated as more severe by patients also treated with SBRT than patients treated with combination therapy only. These findings support the results from the Phase II trial^6^ indicating that the addition of SBRT provides no added clinical benefit for patients receiving treatment for Merkel cell carcinoma who are treated with combined nivolumab and ipilimumab.

This study is characterized by several strengths, including a focus on patients with an understudied cancer diagnosis, a longitudinal study design, and an analytic strategy that identified changes in patient‐reported outcomes during treatment separately from changes after treatment was completed. However, limitations included a small sample size that likely reduced our ability to identify statistically significant differences. Furthermore, the sample was fairly homogenous, such that patients were typically male and White. There was also heterogeneity in the time spent in active treatment such that the timing of the follow‐up assessments relative to treatment initiation was variable across participants. For example, patients who spent more time in active treatment may have had systematically later assessments. As such, these findings should be considered preliminary pending further validation in larger and more diverse patient samples. However, there is limited research on the effects of combination therapy in patients receiving treatment for Merkel cell carcinoma, and little is known about QOL in this population; thus, this study adds critical new information to the existing literature base on patients receiving treatment for Merkel cell carcinoma. Future research on the use of combination therapy in patients with Merkel cell carcinoma should include larger samples to facilitate examination of moderators of change in QOL, including the role of response to treatment and prior receipt of an ICI. In our study, differences in QOL between treatment groups could have been confounded with progressive disease rather than extra toxicity due to SBRT. We hope this study will serve as the basis for future in‐depth studies examining QOL in patients treated with combination therapy.

## 5 CONCLUSION

In conclusion, QOL remained stable over time in patients treated with combined nivolumab and ipilimumab, with improvements in emotional functioning during active treatment. These findings are consistent with prior research on patients treated with single‐agent monotherapies.^8^, ^9^, ^11^ However, our data suggest that QOL may deteriorate during active treatment and post‐treatment in patients also treated with SBRT.

## AUTHOR CONTRIBUTIONS

**Aasha I. Hoogland:** Data curation (equal); formal analysis (lead); visualization (lead); writing – original draft (lead); writing – review and editing (equal). **Andrew S. Brohl:** Resources (equal); writing – original draft (equal); writing – review and editing (equal). **Brent J. Small:** Supervision (equal); writing – review and editing (equal). **Lauren Michael:** Data curation (equal); writing – review and editing (equal). **Evan Wuthrick:** Data curation (equal); writing – review and editing (equal). **Zeynep Eroglu:** Resources (equal); writing – review and editing (equal). **Dukagjin Blakaj:** Resources (equal); writing – review and editing (equal). **Claire Verschraegen:** Resources (equal); writing – review and editing (equal). **Nikhil I. Khushalani:** Resources (equal); writing – review and editing (equal). **Heather S. L. Jim:** Conceptualization (lead); writing – review and editing (equal). **Sungjune Kim:** Conceptualization (supporting); funding acquisition (equal); resources (equal); writing – review and editing (equal).

## FUNDING INFORMATION

This work was supported by the Bristol Myers Squibb Rare Population Malignancy Program. The funder had no role in the study design, data collection, data analysis, data interpretation, writing of the report, or the decision to submit the article for publication.

## CONFLICT OF INTEREST STATEMENT

NIK reports consulting fees from BMS, Merck, Jounce, Novartis, Regeneron, Genzyme, Iovance, Castle Biosciences, Nektar, Replimmune, and Instil Bio; research funding (to institute) from BMS, Merck, Celgene, Novartis, GSK, HUYA, Regeneron, Replimmune, Modulation Therapeutics; participation on a data safety monitoring board with Incyte, AstraZeneca; participation in a study steering committee with BMS, Nektar, Regerenon, Replimmune; and ownership of common stock in Bellicum, Amarin, and Asensus Surgical. SK reports research support from Bristol Myers Squibb for the submitted work and research support from Bristol Myers Squibb and AstraZeneca outside the submitted work. HJ reports being a consultant for SBR Bioscience, grant funding from Kite Pharma. All other authors have no interests to declare.

## Supporting information


Appendix S1.

## Data Availability

Data are available upon reasonable request.
