# Supplementary material for: Quality of life and patient‐reported toxicities in patients with advanced Merkel cell carcinoma treated with combined nivolumab and ipilimumab with or without stereotactic body radiation therapy
Source: Cancer Med. 2024 Jul 17;13(14):e7464. doi: 10.1002/cam4.7464 (PMC11255021; doi:10.1002/cam4.7464)
Supplement: Supplementary file 1 — Appendix S1. [file CAM4-13-e7464-s001.docx]

**Supplemental Table 1.** Parameter estimates from piecewise random-effects mixed models examining changes in quality of life in all randomized participants during active treatment and the post-treatment period.

|  | Overall Quality of Life | Physical Functioning | Role Functioning | Emotional Functioning | Cognitive Functioning | Social Functioning |
| --- | --- | --- | --- | --- | --- | --- |
| Intercept | 65.66*** | 76.33*** | 72.09*** | 77.76*** | 83.52*** | 76.61*** |
| Active treatment | -<0.01 | -<0.01 | -<0.01 | .03* | 0.01 | <0.01 |
| Post-treatment | -0.06 | -0.05 | -0.05 | 0.01 | -0.08** | -0.12** |

**Supplemental Table 2.** Parameter estimates from piecewise random-effects mixed models examining differences in changes in quality of life by treatment arm (combined nivolumab and ipilimumab only vs. combined nivolumab and ipilimumab plus SBRT) during active treatment and the post-treatment period.

|  | Overall Quality of Life | Physical Functioning | Role Functioning | Emotional Functioning | Cognitive Functioning | Social Functioning |
| --- | --- | --- | --- | --- | --- | --- |
| Intercept | 71.44*** | 77.82*** | 78.64*** | 80.33*** | 84.63*** | 78.82*** |
| Treatment arm | -11.12 | -1.88 | -11.99 | 4.86 | -1.68 | 3.55 |
| Active treatment | <0.01 | 0.02 | 0.02 | 0.04* | 0.03 | 0.03 |
| Post-treatment | 0.01 | <0.01 | 0.04 | 0.06 | -0.02 | 0.02 |
| Active treatment x Treatment arm | -0.03 | -0.08*** | -0.08* | -0.02 | -0.04 | -0.08* |
| Post-treatment x Treatment arm | -0.13 | -0.10* | -0.18* | -0.08 | -0.12 | -0.27** |

**Supplemental Table 3.** Patient-rated and clinician-rated symptom severity in patients treated with combined nivolumab and ipilimumab only (n=25).

|  | None | | A little | | Quite a bit | | Very much | |
| --- | --- | --- | --- | --- | --- | --- | --- | --- |
|  | Patients | Clinicians | Patients | Clinicians | Patients | Clinicians | Patients | Clinicians |
| Fatigue | 2 (8) | 6 (24) | 13 (52) | 18 (72) | 4 (16) | 1 (4) | 6 (24) | 0 |
| Nausea | 15 (60) | 16 (64) | 7 (28) | 9 (36) | 2 (8) | 0 | 1 (4) | 0 |
| Vomiting | 18 (72) | 21 (84) | 7 (28) | 4 (16) | 0 | 0 | 0 | 0 |
| Dyspnea | 11 (44) | 21 (84) | 10 (40) | 4 (16) | 4 (16) | 0 | 0 | 0 |
| Diarrhea | 12 (48) | 15 (60) | 9 (36) | 9 (36) | 4 (16) | 1 (4) | 0 | 0 |

Note: “None” corresponds to “not at all” on the patient-reported EORTC-QLQ-C30 and no reported adverse event on the clinician-rated CTCAE. “A little” on the EORTC-QLQ-C30 corresponds to grade 1 or 2 on the CTCAE. “Quite a bit” on the EORTC-QLQ-C30 corresponds to grade 3 on the CTCAE. “Very much” on the EORTC-QLQ-C30 corresponds to grade 4 on the CTCAE.

**Supplemental Table 4.** Patient-rated and clinician-rated symptom severity in patients treated with combined nivolumab and ipilimumab plus SBRT (n=25).

|  | None | | A little | | Quite a bit | | Very much | |
| --- | --- | --- | --- | --- | --- | --- | --- | --- |
|  | Patients | Clinicians | Patients | Clinicians | Patients | Clinicians | Patients | Clinicians |
| Fatigue | 1 (4) | 12 (48) | 7 (28) | 13 (52) | 13 (52) | 0 | 4 (16) | 0 |
| Nausea | 14 (56) | 21 (84) | 8 (32) | 7 (28) | 3 (12) | 0 | 0 | 0 |
| Vomiting | 22 (88) | 22 (88) | 3 (12) | 3 (12) | 0 | 0 | 0 | 0 |
| Dyspnea | 11 (44) | 20 (80) | 6 (24) | 5 (20) | 5 (20) | 0 | 3 (12) | 0 |
| Diarrhea | 13 (52) | 13 (52) | 6 (24) | 11 (44) | 4 (16) | 1 (4) | 2 (8) | 0 |

Note: “None” corresponds to “not at all” on the patient-reported EORTC-QLQ-C30 and no reported adverse event on the clinician-rated CTCAE. “A little” on the EORTC-QLQ-C30 corresponds to grade 1 or 2 on the CTCAE. “Quite a bit” on the EORTC-QLQ-C30 corresponds to grade 3 on the CTCAE. “Very much” on the EORTC-QLQ-C30 corresponds to grade 4 on the CTCAE.
